# Construction of a new tumor immunity-related signature to assess and classify the prognostic risk of ovarian cancer

**DOI:** 10.18632/aging.103868

**Published:** 2020-11-08

**Authors:** Jiashan Ding, Qiaoling Zhang, Shichao Chen, Huikai Huang, Linsheng He

**Affiliations:** 1 Obstetrics and Gynecology, The First Affiliated Hospital of Nanchang University, Nanchang University, Nanchang, Jiangxi, China

**Keywords:** ovarian cancer, antigen processing and presentation, signature, nomogram, DCA

## Abstract

Ovarian cancer is associated with a high mortality rate. In this study, we established a new immune-related signature that can stratify ovarian cancer patients. First, we obtained immune-related genes through IMMUPORT, and DEGs (Differential Expression Genes) by analyzing the GSE26712 dataset. The APP (Antigen Processing and Presentation) and DEG signatures were established using univariate and multivariate Cox models. Kaplan-Meier analysis revealed the signatures’ prognostic value in training and validation cohorts (HR: 0.379 VS. 0.450; 0.333 VS. 0.327). Nomogram analysis was used to assess the signatures’ ability to predict the 30-month prognosis, which was evaluated using the calibration curve and time-dependent ROC curve (30-month AUC: 0.665 VS. 0.743). Time-dependent ROC, Decision Curve Analysis (DCA) and Integrated discrimination improvement (IDI) was used to compare the new model to previously published gene signatures. 30-month AUC composite variable (0.736) was higher than 9-gene signature (0.657), and composite variable had a larger net benefit and a higher IDI (+2.436%) relative to the 9-gene signature. Tumor immune infiltration and tumor microenvironment scores of the 2 groups separated by APP signature were compared. GSEA was used to identify enriched KEGG pathways. Conclusively, the proposed signature can stratify ovarian cancer patients by risk-score and guide clinical decisions.

## INTRODUCTION

Ovarian cancer is one of the malignant tumors of the female reproductive system, characterized by a high mortality rate. With the deepening of research on tumor molecular mechanisms, for example, N7-Methyl-2'-deoxyguanosine (m7dG) affects DNA replication by slowing down the catalytic efficiency of DNA polymerase β [1], and the research of new antitumor drugs, for example, the method innovation of synthesis of Solasodine acetate [2], an anticancer steroidal alkaloid, the mechanism of human tumor is becoming gradually clear and the overall survival rate also proves be improved. However, pathogenesis remains unclear of ovarian cancer. A dualistic origin for high grade serous ovarian carcinoma (HGSOC) indicates that HGSOC may originate from both fallopian tube (FTE) and ovarian surface epithelium (OSE), and this may influence its therapeutic response [3]. Between 1995-2014, remarkable progress has been made in OC treatment, enhancing survival while lowering mortality and incidence in the 19 jurisdictions included in the study [4]. Minor reductions in disease-specific mortality are attributable to the use of oral contraceptives, recent expansion of risk-reducing surgery among women from high-risk genetic backgrounds, and a reduction in long-term hormone replacement [5]. On the contrary, a prospective study indicated that elevated levels of circulating sphingomyelins, 3-23 years before diagnosis were associated with lower OC risk, regardless of histotype, with stronger associations among postmenopausal women [6]. Although OC overall survival (OS) and progression-free interval (PFI) have improved, many clinical challenges remain, including resistance to cisplatin chemotherapy, tumor recurrence, and late diagnosis [7]. Highlighting the need for improved understanding of OC and identification of novel biomarkers. Such biomarkers may facilitate patient’s stratification for improved outcomes.

The main treatment for OC is surgery combined with platinum drugs, or paclitaxel chemotherapy [8]. However, recent studies have highlighted the potential benefits of immunotherapy against various cancers, including OC. Therapeutic targeting of T cell inhibitory checkpoint proteins CTLA-4 and PD(L)1 is efficacious in many cancers, reducing tumor burden and increasing long-term survival [9]. Combining immuno-modulatory agents with low-dose whole-abdominal radiotherapy may enhance activity of beneficial immune cells while blocking or reprograming inhibitory ones such as MDSCs, M2 macrophages, and Tregs. These radiotherapy modalities represent new opportunities in OC treatment, promising to enhance the efficacy of immunotherapy against this disease [10]. Immunotherapy is associated with side effects, including skin toxicity and resistance [11, 12]. Here, we aimed to identify novel immune-related signatures that can stratify OC patients into high and low risk groups and identify those likely to benefit most from immunotherapy. We further explored the relationship between the new signatures, immune cell tumor infiltration and the tumor microenvironment (tumor stroma and immune components).

## RESULTS

### Construction of an immune-related prognostic risk model

**Figure 1:**
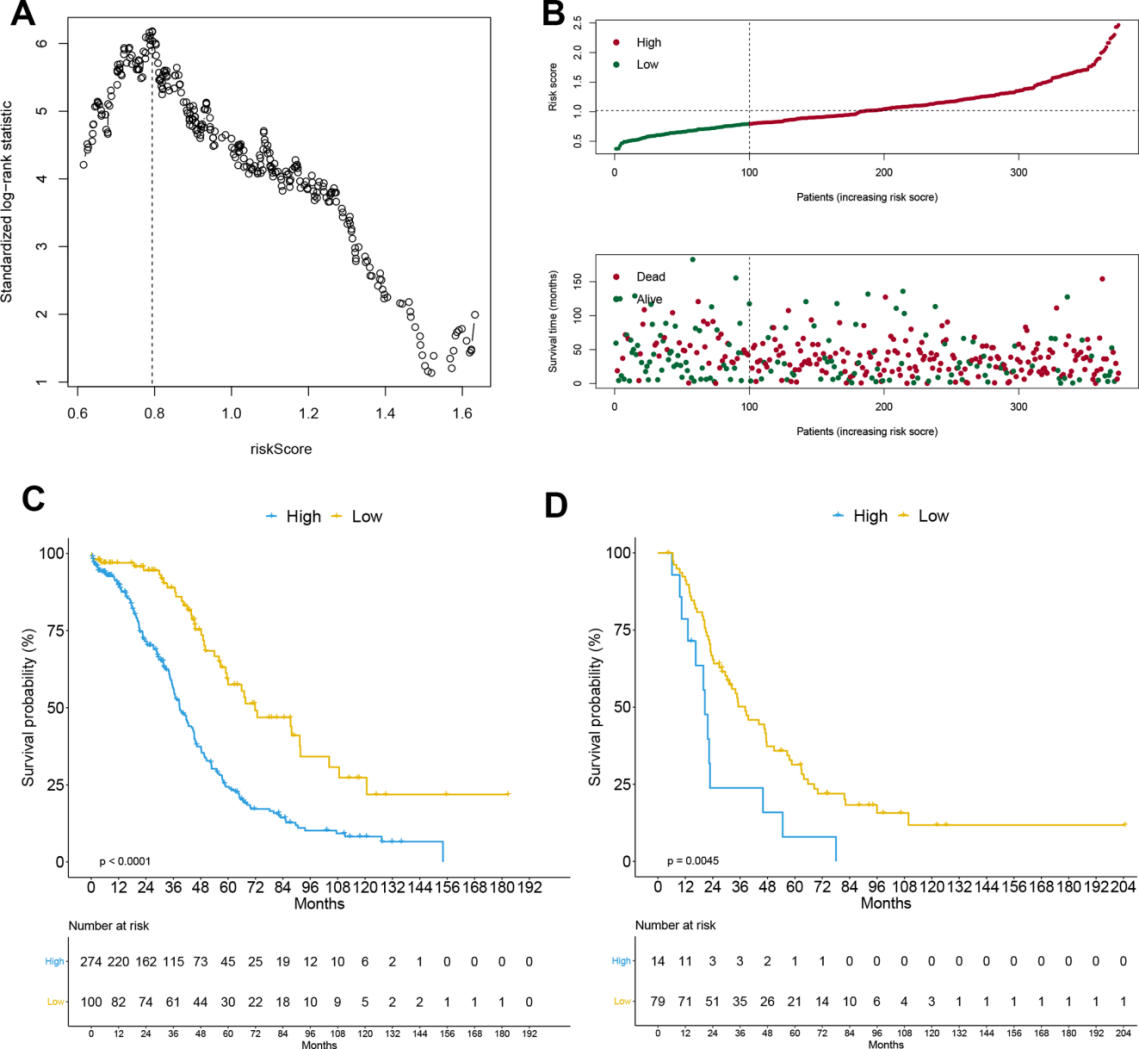
**Construction of APP signature.** The optimal cutoff calculated by the maximally selected rank statistics (**A**), risk score analysis (**B**) including risk curve and distribution map of survival status in the training cohort, and the Kaplan-Meier curve grouped by APP signature in training cohort (**C**) and external validation cohort (**D**).

We found 148 antigen processing and presentation (APP) genes from the IMMUNE database. Of these, 11 were identified by univariate Cox model (*P* <0.05): HLA-A, HLA-DOB, HLA-F, HSPA1L, IFNγ, LTA, PSMB8, PSMC1, PSME2, TAP1 and UBR1. Thus, a multivariate Cox regression model was performed using 11 antigen-presenting-related genes (Table 1), and the APP score calculated using the formula mentioned above. Scores were separated using the best statistical cutoff of 0.794 established by maximally selected rank statistics (Figure 1A). Patients with APP scores lower than the cutoff were categorized in “low” group. The rest were grouped into the “high” group. The prognostic features of the 2 groups were evaluated in the training dataset using survival curves and Kaplan-Meier analysis (Figure 1B, 1C). OS was significantly lower for OC patients in the “high” group relative to the “low” group (HR = 0.379, 95% CI = (0.269, 0.534), *p* = 3.05e-08). Next, multivariate Cox model analysis on the training data using stage, age, and APP signature revealed that APP signature was independently prognostic in OC (HR = 0.41026, 95% CI = (0.291, 0.579), *p* = 4.13e-07). Patient age also emerged as an independent prognostic factor (HR= 1.000, 95% CI = (1.000, 1.000), *p* = 0.001). Suggesting that APP signature can stratify patients by OS. Next, a similar analysis was done on the validation dataset to validate the prognostic role of the APP signature (HR = 0.4242, 95% CI = (0.231, 0.781), *p* = 0.005, for univariate Cox regression model) (Figure 1D). Multivariate Cox analysis (age, stage and APP signature) revealed that the low APP signature was an independent prognostic factor of protection (HR = 0.450, 95% CI = (0.243, 0.832), *p* = 0.011). In contrast, age and disease stage are not independent prognostic factors in OC.

**Table 1:** Univariate and multivariate Cox analysis of antigen-presenting-related genes.

**Genesymbol**	**Univariate Cox regression model**						**Multivariate Cox regression model**				
	**coef**	**HR**	**HR.95L**	**HR.95H**	**P value**		**coef**	**HR**	**HR.95L**	**HR.95H**	**P value**
HLA-A	-0.12219	0.884978	0.784906	0.997809	0.045958		0.227042	1.254883	0.927314	1.698163	0.141282
HLA-DOB	-0.14834	0.862136	0.796184	0.933551	0.000259		-0.15474	0.856641	0.751962	0.975892	0.019968
HLA-F	-0.11502	0.891353	0.810994	0.979674	0.017034		-0.10184	0.903173	0.732728	1.113266	0.339875
HSPA1L	-0.18284	0.832899	0.700867	0.989804	0.037862		-0.14903	0.86154	0.709874	1.045609	0.131421
IFNG	-0.0774	0.925521	0.861848	0.993899	0.033317		-0.04107	0.959764	0.858457	1.073027	0.470564
LTA	-0.09626	0.908228	0.835783	0.986954	0.023232		0.021196	1.021422	0.886346	1.177083	0.769615
PSMB8	-0.10547	0.899903	0.810651	0.998982	0.047808		0.26473	1.30308	0.977904	1.736383	0.070697
PSMC1	-0.2849	0.752087	0.580835	0.973829	0.030685		-0.22198	0.800935	0.605311	1.059782	0.120284
PSME2	-0.17636	0.838316	0.719252	0.97709	0.024039		-0.08598	0.917616	0.72087	1.168059	0.484996
TAP1	-0.13047	0.877686	0.79804	0.965281	0.007188		-0.21124	0.809576	0.632694	1.03591	0.09306
UBR1	0.358284	1.430872	1.102333	1.857329	0.007102		0.316856	1.372804	1.030578	1.828675	0.030323

### Construction of a differential gene-related prognostic risk model

**Figure 2:**
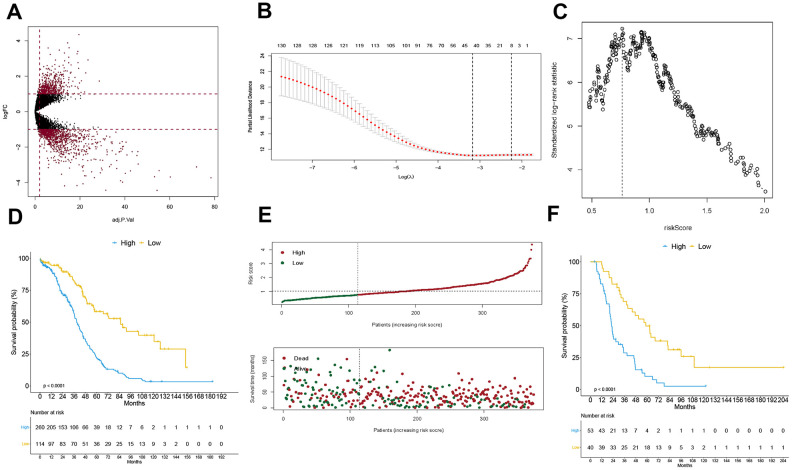
**Construction of DEG signature.** Volcano plot for displaying differentially expressed genes in GSE26712 cohort (**A**), the procedure of data dimension reduction by lasso algorithm to select the optimal candidate genes (**B**), and the optimal cutoff of DEG signature calculated by the maximally selected rank statistics (**C**), the Kaplan-Meier curve grouped by DEG signature in training cohort (**D**) and external validation cohort (**F**), and the risk score analysis in the training cohort (**E**).

The GSE26712 dataset, which comprised of 10 normal ovarian epithelial tissues and 185 OC tissues, was used for analysis of differentially expressed genes (DEGs). This analysis identified 1540 DEGs, of which 973 had low expression and 567 high expression (Figure 2A). The univariate Cox model identified 130 genes associated with prognosis (*p* <0.05) (Supplementary Table 1). These were further analyzed with LASSO to reduce the dimensionality of values (Figure 2B). After cross validation, the optimal lambda value and candidate genes were identified, at which the minimal mean squared error, and candidate genes were used to calculate DEG score by establishing the multivariate Cox model. Next, we used maximally selected rank statistics to calculate an optimal cutoff value of 0.764 (Figure 2C), which was used to stratify OC patients. Those scoring <0.764 were classified in the “low” group, which was considered to have good prognosis. The rest were placed in the “high” group, and had poor prognosis, as revealed by Kaplan-Meier analysis (HR = 0.333, 95% CI = (0.240, 0.463), *p* = 5.89e-11) (Figure 2D–2E). The multivariate Cox model (DEG score, age and stage) demonstrated that the DEG signature and age were independent factors (P<0.05). As showed in Supplementary Table 1, “low” group could be acted as independent prognostic protective factor (HR= 0.282, 95% CI= (0.200, 0.398), P= 7.34e-13); age also was a prognostic risk factor (HR= 1.000, 95% CI= (1.000, 1.000), P= 2.59e-05). In the external validation cohort, the DEG signature could distinguish patients with different prognosis. Relative to the “low” group, OS of patients in the “high” group was significantly worse (HR = 0.327, 95% CI = (0.199, 0.537), *p* = 9.78e-06) (Figure 2F). The lower DEG score was an independent prognostic protective factor for OC (HR = 0.309, 95% CI = (0.179, 0.533), *p* = 2.44e-05), although this was not statistically significant for age and disease stage as showed in multivariate Cox analysis.

### Composite variable, including APP signature and DEG signature, improved patient prognosis estimation

**Figure 3:**
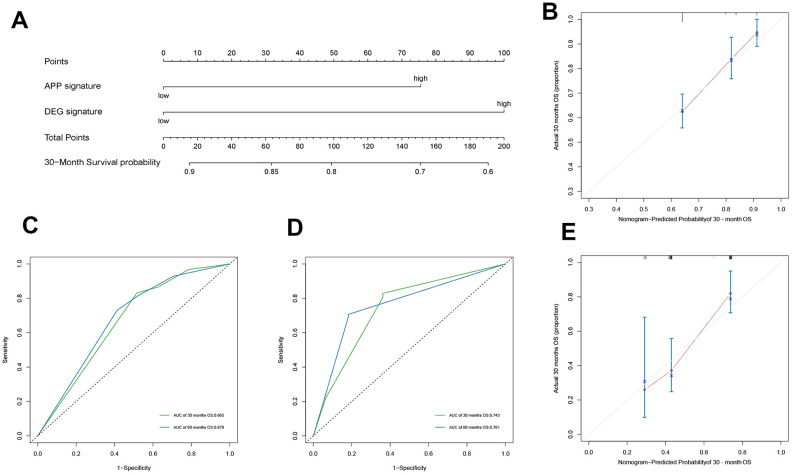
**Construction of nomogram and evaluation of its prediction ability.** The nomogram plot (**A**) composed of APP signature and DEG signature, the calibration curve for the estimation of 30-month survival probability in the training cohort (**B**) and the validation cohort (**C**), and the time-dependent ROC of 30 months describe the discriminative ability of the nomogram in the training cohort (**D**) and the validation cohort (**E**).

The APP and DEG signatures were independent of each other as revealed by multivariate Cox regression analysis established by the 2 variables (HR = 0.507, 95% CI = (0.354, 0.726), *p* = 0.0002; HR = 0.406, 95% CI = (0.288, 0.574), *p* = 3.12e-07). Other clinical variables, including age and disease stage were excluded from this analysis because of their small contributions. Based on the multivariate Cox regression analysis, a nomogram, a quantitative method to predict individual probability of overall survival, was established to estimate OC 30-month OS (Figure 3A). The prediction values of the 30-month nomogram in the calibration plot were very close to the 45-degree line in the training and validation datasets (Figure 3B–3C), indicating the consistency of the model. Time-dependent ROC is shown in Figure 3D–3E. The 30-month AUCs were 0.665 and 0.743, and the 60-month AUCs were 0.679 and 0.761.

### Comparison with previously published gene signatures

**Figure 4:**
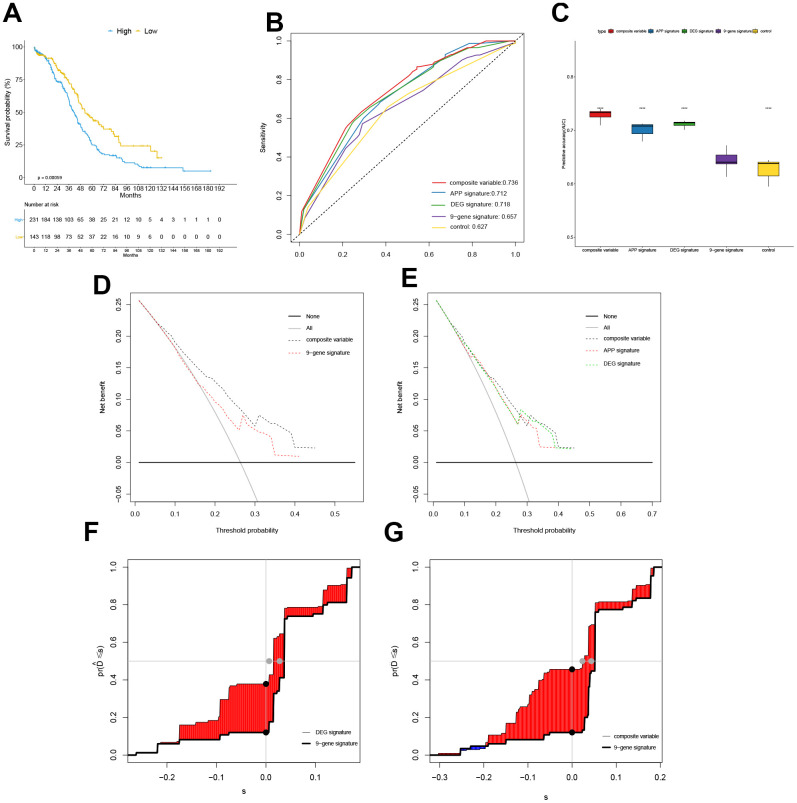
**Comparison with previously published gene signatures.** The Kaplan-Meier curve grouped by 9-gene signature in training cohort (**A**), the time-dependent ROC at 30 months in the TCGA cohort (**B**), the box plot for comparing AUC values of different models ("****" means P value <0.0001) (**C**), Decision Curve Analysis (DCA) (**D**–**E**), and the plot of Integrated Discrimination Improvement (IDI) (**F**–**G**).

Based on a 9-gene signature constructed by Tzu-Pin Lu and Kuan-Ting Kuo et al. [13] to predict OC OS, we establish a multivariate Cox regression model to calculate risk score and construct a signature named 9-gene signature. The best cutoff of 0.926 calculated by the maximally selected rank statistics was used to convert the continuous scores to binary ones. Kaplan-Meier analysis revealed that OC patients in the “high” group had a poor prognosis relative to the low group (Figure 4A). Cox models were established using APP signature, DEG signature, composite variable including APP signature and DEG signature, age, stage and 9-gene signature, and the discriminative abilities of the models examined by time-dependent ROC curve and its AUCs (Figure 4B). The 30-month AUCs of composite variable, APP signature, and DEG signature were 0.736, 0.712, and 0.718, respectively, which was better than the 9-gene signature (AUC=0.657) and control model (AUC=0.627) containing age and stage only. Upon performance of 1000 resampling (the number of resampling seeds was set to 42), using the "bootstrap" method, the 30-month AUCs of APP signature, DEG signature, composite variable was significantly higher than that of the 9-gene signature (p<0.001). However, the 9-gene signature was better than the control model (p <0.01) (Figure 4C). Decision Curve Analysis (DCA) was used to evaluate clinical net benefit (Figure 4D–4E). Composite variable models provided a larger net benefit relative to the 9-gene signature when the risk threshold probability for a clinician or a patient ranged between 0.6 and 0.45. However, APP signature, DEG signature and composite variable model had a similar net benefit. Integrated discrimination improvement (IDI) was performed using “survIDINRI” R package. Relative to the old 9-gene signature, the discriminative degree of the DEG signature and composite variable were significantly improved (4.822%, 95% CI (0.465%, 9.229%), *p* = 0.020; 6.239%, 95% CI (2.135%, 10.053%, *p* <0.001) (Figure 4F–4G), but the IDI increase in APP signature was not significant (2.436%, 95% CI (-0.706%, 6.103%), *p* =0.119).

### Analysis of immune cell infiltration and GSEA in different subgroups

**Figure 5:**
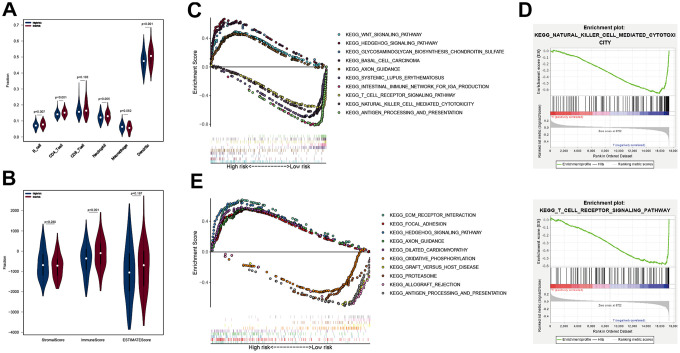
**Analysis of immune cell infiltration and GSEA in different subgroups.** The B cell, CD4 T cell, neutrophil and Dendritic scores of the two subgroups in TCGA cohort (**A**), the StromalScore, ImmunesSore, and ESTIMATEScore of the two subgroups in TCGA cohort (**B**); ten KEGG pathways analyzed by GSEA (**C**), in which groups was separated by APP signature, and two immune-related KEGG pathways (**D**); ten KEGG pathways analyzed by GSEA (**E**), in which groups was grouped by DEG signature.

The “immuneEstimation” data, calculated from RNA-Seq expression profiles using TIMER algorithm was analyzed to establish the abundance of 6 tumor-infiltrating immune cells (TIICs). This analysis identified 367 cases of immune cell infiltration in OC. Next, violin plots were used to compare the abundance of 6 TIICs in OC, and groups defined based on APP signature (Figure 5A). B cell, CD4 T cell, neutrophil and dendritic cell scores were higher in the low group relative to the high group (*p* = 0.007, <0.001, 0.005 and <0.001) (Figure 5B). However, CD8 T cell and macrophage scores were not significantly different between the 2 groups, indicating that good prognosis associated with the low group may result from higher infiltration by B cells, CD4 T cells, neutrophils and dendritic cells. Next, immune and stromal scores were calculated using the“estimate” package on R, and divided into 2 groups by APP signature. Similarly, immune scores in the low group were higher than in the high group (*p* = 0.001). There were no significant differences between stromal cell scores and total scores.

Because there were no immune-related, as well as up-regulated pathways in the high group, we selected the top 5 pathways based on the enrichment score and along with them, displayed the 5 immune-associated and up-regulated pathways in the low group (Figure 5C). The 5 immune-associated pathways were associated with antigen processing and presentation, natural killer cell mediated cytotoxicity, T cell receptor signaling pathway, intestinal immune network for IGA production and systemic lupus erythematosus (Figure 5D). The 5 upregulated pathways in the high group were associated with WNT signaling pathway, hedgehog signaling pathway, glycosaminoglycan biosynthesis chondroitin sulfate, basal cell carcinoma and axon guidance. This result was consistent with the above conclusion. The lower APP signature may increase tumor immune cell infiltration by up-regulating immune-related pathways, improving outcomes. The 10 KEGG pathways associated with DEG signature were displayed in Figure 5E. It was worth noting that the "ANTIGEN_PROCESSING_AND_PRESENTATION" KEGG pathway was enriched by the DEG signature low-risk group, which was consistent with the previous results. Reasonably, the good OS of the DEG signature low-risk group may be related to the tumor immune response, but its specific mechanism remained to be further studied.

## DISCUSSION

Antigen-presenting cells (APC) include dendritic cells, macrophages, and B lymphocytes. The ability to recognize, present, and process tumor antigens is a critical factor affecting cancer prognosis. Breast cancer studies have shown that differences in antigen processing and presentation caused differential immune-mediated antitumor responses activated by radiation [21]. Some research is devoted to the research and development of cancer vaccines. Nanoparticles, an adjuvant for cancer vaccines, come into people's vision, which can enhance antigen presentation and stimulate immune responses [22]. CD44-targeted PLGA nanoparticles can be a carrier of chemotherapy drugs and siRNA and selectively delivered them to the corresponding targets to improve OC chemotherapy resistance [23]. Additionally, cytokines regulate the balance between pro- and anti-tumor immunity. Local tumor-associated macrophages activated by T-cells, IFN-γ and GM-CSF may improve antigen processing and presentation by host macrophages to antigen-specific T cells. Activation of host macrophages transforms the TME from immunosuppressive to immunostimulatory and has anti-OC effects [24]. We identified 11 antigen presentation and processing genes (HLA-A, HLA-DOB, HLA-F, HSPA1L, IFNγ, LTA, PSMB8, PSMC1, PSME2, TAP1 and UBR1) related to OC prognosis upon TCGA data analysis, consistent with previous studies. HLA-A genotypes are valuable prognostic biomarkers in epithelial OC, and their downregulation is associated with poor survival [25]. Furthermore, downregulated IFNγ was also associated with poor OC survival [26]. Here, we uncovered DEGs in OC and ovarian epithelial tissues, identified those with prognosis value, and reduced the number of candidate genes by LASSO regression analysis. Surprisingly, the DEG signature performed very well on the trainingand validation datasets, and can stratify OC patients into high- and low-risk groups. Relative to APP signature, the DEG signature may have superior OC stratification capacity.

Although bevacizumab has been used in advanced OC treatment alone or in combination with Olaparib with substantial benefit in OC patients with homologous recombination deficiency, including those without a BRCA mutation [27, 28], late stage disease is associated with a 29% 5-year survival rate [29]. The improvement of OC overall survival rate, especially in advanced OC is a common problem. However, the pursuit of precise treatment is a promising entry point. Effective stratification of OC patients may identify those likely to benefit from chemotherapy, targeted agents, or immunotherapy [30, 31]. MiROvaR, a microRNA-based signature, that can classify OC patients into 2 groups by prognostic value [32]; Manuela Tumiati established a functional homologous recombination assay to predict primary chemotherapy response and long-term survival in OC patients [33]. An immune-related gene signature for OC is lacking. Thus, the signature we have established may identify tumor immune deficiency populations who may benefit from immunotherapy, which is critical for clinicians and patients making treatment choices.

However, development of biomarkers that can predict responsiveness to various immunotherapies may allow better treatment selection. Therefore, OC immunotherapies have to take into account immune suppressive networks within the TME [34]. Infiltration by immune cells, and activated T cell recruitment to tumor sites are critical for tumor immunity as they affect tumor cell killing [35]. In lung cancer, Treg and Th17 cells in the TME modulate cytokine and chemokine production, promote immune cell recruitment and help regulate anti-tumor and pro-tumor immune cell activation states [36]. However, T-cell infiltration can be impeded by local TME factors, including dense stroma, aberrant vasculature, and immunosuppressive factors such as TGFβ, which is an immunosuppressive cytokine that inhibits T-cell effector function by inhibiting antigen-presenting DCs [37]. Based on the TCGA transcriptome data, we obtained data on immune cells abundance in OC tissue and scored the TME, proving that there is a significant difference in tumor immune cell infiltration and tumor purity in different immune risk groups. The low-risk group of APP signature, including T cells, NK cells, and dendritic cells, has a higher degree of immune cells infiltration relative to the high-risk group. Tumor cells purity in the low-risk group is lower than in the high-risk group. GSEA enrichment analysis found that T-cells and NK cell-related KEGG pathways were upregulated in the low-risk group. These results indicate that APP signature may reflect tumor immunity in OC patients well, which may be a valuable reference for clinical immunotherapy.

Our study identified new gene signatures. These signatures can identify high and low-risk OC patients, provide valuable clinical guidance for immunotherapy, and provide references for individualized treatment measures in OC.

## MATERIALS AND METHODS

### Data acquisition and processing

OC transcriptome data were downloaded from TCGA (https://www.cancer.gov/) and ICGC database (https://dcc.icgc.org/). The TCGA dataset comprised of 379 OC samples, of which 2 with incomplete survival data and 3 replicates were excluded. The ICGC dataset had 93 samples with complete follow-up data. The count data of RNAseq of OC was normalized using “limma” package on R, and transformed to log2 scale for further analysis.

### APP signature construction

Given that tumor immunity is closely related to the antigen presentation and processing function of immune cells, 148 antigen-presenting-related genes were obtained from ImmPort (https://www.immport.org/home) to construct univariate Cox model and identify candidate genes for establishing a multivariate Cox model. The multivariate Cox model was used to calculate risk score using the formula; $\sum_{m=1}^{m}\beta_{m}*X_{m}$ (where X is candidate gene expression value, and β is regression coefficient obtained from the multivariate Cox model). Maximally selected rank statistics was used to determine an optimal cutoff value and divide continuous risk scores into binary variables. The risk score model obtained in the training dataset is termed APP signature.

### DEG signature construction

The GSE26712 dataset was downloaded GEO (https://www.ncbi.nlm.nih.gov/gds/) and consisted of 10 normal ovarian epithelial tissues and 185 OC tissues. This dataset was used to identify DEGs in normal vs tumor tissues. Adjusted P (adj. P) values were applied to correct false positive results using default Benjamini-Hochberg false discovery rate (FDR) method [14]. The DEG analysis threshold was set at *adj. P* <0.05 and | log2 FC|>1. This analysis identified 1,540 DEGs. They were then incorporated into the univariate Cox regression model for candidate prognostic DEGs selection. Due to the huge number of DEGs, LASSO algorithm was used to reduce data dimensionality, which narrowed candidate DEGs to 8. Risk score for the 8 candidate DEGs was calculated using multivariate Cox model with the formula $\sum_{m=1}^{m}\beta_{m}*X_{m}$ (where X is candidate gene expression value, and β is regression coefficient obtained from multivariate Cox model). The new risk score model is termed DEG signature. OS was presented as Kaplan-Meier curves in 2 risk score groups that were separated by an optimal cutoff value determined by the maximally selected rank statistics.

### Nomogram construction

Nomograms are designed to answer a specific question and when appropriately used, they are valuable tools for clinicians and patients. They generate an individual’s probability of having a certain clinical outcome by integrating diverse prognostic and determinant variables [15]. Only the APP and DEG signatures were integrated into the “rms” R package to build a multivariate Cox model, because clinical variables like age and stage contributed little to the model compared with APP and DEG signature. Next, a nomogram was constructed to estimate probability of patient survival. Based on the APP and DEG signatures, nomogram scores for all patients could be obtained, as well as the total score (sum of the 2 scores) and survival probability. Considering the consistency between the actual survival and the predicted survival probability of the nomogram, the nomogram predicted the 30-month survival probability after OC diagnosis. A calibration curve was used to describe the actual survival probability and the prediction value of the nomogram on OC patients in the training and the validation datasets. The closeness of bias-corrected lines for both the 30-month and the diagonal line indicated that the model had good consistency [16]. Time-dependent receiver operating characteristic (time-dependent ROC) curve was constructed on the training dataset and the external validation dataset, and the area under curve (AUC) calculated to describe the model’s ability to discriminate.

### Decision curve analysis and integrated discrimination improvement

Decision curve analysis (DCA) assesses model utility in decision making relative to conventional performance measures, such as P values, relative risks, and the concordance index, which cannot indicate whether a model is worth using, which of 2 models is better, or whether data on an additional predictor is necessary [17, 18]. We therefore constructed a DCA to compare clinical net benefit between the APP signature, DEG signature and 9-gene signature published by Tzu-Pin Lu and Kuan-Ting Kuo. The significance of the new signatures on OC prognosis was also evaluated. ROC curve and its AUC were used to compare the differentiating ability of the gene signatures. Additionally, IDI was used to quantitatively evaluate improvements in the diagnostic performance of the new signatures over the 9-gene signature. IDI not only visually displayed the proportion of OC patients correctly reclassified, but also calculated the cost-effectiveness, a function that conventional indicators do not have.

### Tumor immune cell infiltration

Immune cell infiltration data for TCGA tumor samples were obtained from TIMER (https://cistrome.shinyapps.io/timer/) [19], including 6 types of immune cell infiltration data, including B cells, CD4 T cells, CD8 T cells, neutrophils, macrophages and dendritic cells, in more than 20 tumors. Data analysis, including abundance of infiltrating immune cells was done using TIMER algorithm based on RNA-Seq expression profiles data. We matched the samples of the training dataset with the TIMER data, and then got immune cells infiltration data for 367 OC samples. Wilcox non-parametric test was used to compare whether there were differences in the infiltration levels of the 6 immune cells in the 2 groups separated by APP signature.

### Tumor microenvironment

The tumor microenvironment (TME) is the cellular environment in which a tumor exists. Other than tumor cells, the TME comprises of blood vessels, extracellular matrix, stromal cells, fibroblasts, immune cells (including T lymphocytes, B lymphocytes, natural killer cells and natural killer T cells) [20]. Based on TCGA OC transcriptome expression data, we obtained TME scores through the “estimate” R package, including StromalScore, ImmunesSore, and ESTIMATEScore. The ESTIMATE algorithm performs single sample Gene Set Enrichment Analysis to predict tumor purity. The scores were divided into 2 groups by APP signature, and the score differences between the groups compared by Wilcox test.

### GSEA

GSEA (Gene Set Enrichment Analysis) was performed to identify KEGG pathways with *P* <0.05 and FDR <0.25 between the 2 groups. First, OC expression data was divided into high group and low group by APP signature DEG signature, respectively, and KEGG pathway enrichment analysis done using GSEA 4.0 software. The top-20 upregulated pathways were selected in 2 groups based on enrichment analysis score. The focus of this study was to assess the relationship between tumor immunity and OC prognosis. Therefore, all immune-related pathways were extracted from these 40 pathways to elucidate underlying mechanisms on how immunity affects tumors.

### Statistical analysis

All statistical analyses were done using R (https://www.r-project.org/). GSEA software (https://www.gsea-msigdb.org/gsea/index.jsp) was for gene enrichment analysis. Survival data are presented as Kaplan-Meier curves, and comparison between groups done by log-rank test. Time-dependent ROC curve and its AUC were used to describe the prognostic models’ discriminative abilities. IDI value was also used to compare distinguishing abilities between different models. *P* = <0.05 indicated statistical significance.

## Supplementary Material

Supplementary Table 1
