# Supplementary material for: Construction of a new tumor immunity-related signature to assess and classify the prognostic risk of ovarian cancer
Source: Aging (Albany NY). 2020 Nov 8;12(21):21316–28. doi: 10.18632/aging.103868 (PMC7695433; doi:10.18632/aging.103868)
Supplement: Supplementary Table 1 [file aging-12-103868-s001..docx]

**Supplementary Table 1. Univariate and multivariate Cox analysis of DEGs.**

| genesymbol | Univariate Cox regression model | | | | |  | Multivariate Cox regression model | | | | |
| --- | --- | --- | --- | --- | --- | --- | --- | --- | --- | --- | --- |
|  | coef | HR | HR.95L | HR.95H | P value |  | coef | HR | HR.95L | HR.95H | P value |
| AADAC | -0.13752 | 0.871521 | 0.816984 | 0.929699 | 3.03E-05 |  | -0.12051 | 0.886465 | 0.823794 | 0.953904 | 0.001275 |
| ACTN4 | 0.179402 | 1.196502 | 1.003435 | 1.426716 | 0.045699 |  | - | - | - | - | - |
| ADCY9 | 0.197233 | 1.218028 | 1.049214 | 1.414004 | 0.009567 |  | - | - | - | - | - |
| ADH1C | 0.071954 | 1.074606 | 1.008759 | 1.144751 | 0.025729 |  | - | - | - | - | - |
| AFF1 | 0.21483 | 1.239652 | 1.016851 | 1.511269 | 0.033563 |  | - | - | - | - | - |
| AKAP11 | 0.307524 | 1.360054 | 1.074958 | 1.720763 | 0.010402 |  | - | - | - | - | - |
| AMMECR1 | -0.24848 | 0.779983 | 0.663995 | 0.916232 | 0.002486 |  | -0.12844 | 0.879466 | 0.7322 | 1.05635 | 0.169544 |
| ARID5B | 0.217029 | 1.24238 | 1.03325 | 1.493839 | 0.021012 |  | - | - | - | - | - |
| ARL6IP5 | -0.23408 | 0.7913 | 0.653245 | 0.958532 | 0.016715 |  | - | - | - | - | - |
| ATRX | -0.25699 | 0.773377 | 0.598553 | 0.999263 | 0.049345 |  | - | - | - | - | - |
| BLOC1S1 | -0.20698 | 0.813037 | 0.701534 | 0.942262 | 0.005956 |  | - | - | - | - | - |
| BNC1 | 0.058592 | 1.060343 | 1.008909 | 1.114398 | 0.02091 |  | - | - | - | - | - |
| BRD1 | 0.219616 | 1.245598 | 1.001194 | 1.549665 | 0.048762 |  | - | - | - | - | - |
| BTN3A3 | -0.21331 | 0.807906 | 0.704938 | 0.925914 | 0.002166 |  | -0.14833 | 0.862144 | 0.725743 | 1.024182 | 0.091402 |
| C4BPA | -0.05703 | 0.944566 | 0.893097 | 0.999001 | 0.046053 |  | - | - | - | - | - |
| C6orf120 | 0.201333 | 1.223033 | 1.021636 | 1.464131 | 0.028298 |  | - | - | - | - | - |
| CBX7 | 0.190666 | 1.210055 | 1.038274 | 1.410258 | 0.014656 |  | - | - | - | - | - |
| CCDC93 | 0.270761 | 1.310962 | 1.040327 | 1.652002 | 0.021729 |  | - | - | - | - | - |
| CCT5 | -0.24213 | 0.784956 | 0.621142 | 0.991971 | 0.042615 |  | - | - | - | - | - |
| CERK | 0.235664 | 1.265749 | 1.064263 | 1.505381 | 0.007721 |  | - | - | - | - | - |
| CFI | -0.14462 | 0.865351 | 0.767429 | 0.975768 | 0.018259 |  | - | - | - | - | - |
| CIC | 0.181051 | 1.198477 | 1.012081 | 1.419202 | 0.0358 |  | - | - | - | - | - |
| CIZ1 | 0.352964 | 1.42328 | 1.108485 | 1.827473 | 0.005648 |  | - | - | - | - | - |
| CX3CR1 | 0.122517 | 1.130338 | 1.045424 | 1.222149 | 0.002106 |  | 0.099059 | 1.104132 | 1.012572 | 1.203971 | 0.024907 |
| CXCL13 | -0.08406 | 0.919379 | 0.875589 | 0.965358 | 0.000736 |  | -0.07626 | 0.926573 | 0.874878 | 0.981322 | 0.009223 |
| CXCR4 | -0.14643 | 0.863789 | 0.765998 | 0.974065 | 0.016912 |  | - | - | - | - | - |
| DEK | -0.22367 | 0.799582 | 0.660544 | 0.967886 | 0.021741 |  | - | - | - | - | - |
| DGKZ | 0.283991 | 1.328421 | 1.040289 | 1.696358 | 0.02281 |  | - | - | - | - | - |
| DLG5 | 0.143679 | 1.154513 | 1.012196 | 1.316841 | 0.032309 |  | - | - | - | - | - |
| DOCK4 | 0.144804 | 1.155813 | 1.00899 | 1.324 | 0.0367 |  | - | - | - | - | - |
| DPYS | -0.09386 | 0.910412 | 0.842001 | 0.984381 | 0.018526 |  | - | - | - | - | - |
| ECI2 | -0.25747 | 0.773002 | 0.65163 | 0.916979 | 0.003132 |  | - | - | - | - | - |
| ELP1 | 0.277302 | 1.319565 | 1.01663 | 1.712767 | 0.037168 |  | - | - | - | - | - |
| EPB41L2 | 0.20153 | 1.223272 | 1.075877 | 1.390861 | 0.002095 |  | - | - | - | - | - |
| EPCAM | -0.17177 | 0.842177 | 0.719649 | 0.985565 | 0.032257 |  | - | - | - | - | - |
| EPS8 | 0.147462 | 1.15889 | 1.010908 | 1.328533 | 0.034378 |  | - | - | - | - | - |
| ERBB2 | 0.270879 | 1.311116 | 1.10283 | 1.558741 | 0.002149 |  | - | - | - | - | - |
| EXOSC4 | -0.14626 | 0.863931 | 0.747929 | 0.997923 | 0.046787 |  | - | - | - | - | - |
| FAM8A1 | -0.23825 | 0.788002 | 0.643039 | 0.965644 | 0.021618 |  | - | - | - | - | - |
| FANCI | -0.17372 | 0.840529 | 0.715832 | 0.986949 | 0.033981 |  | - | - | - | - | - |
| FKBP11 | -0.21545 | 0.806178 | 0.663443 | 0.979623 | 0.030232 |  | - | - | - | - | - |
| FLOT1 | -0.2033 | 0.816032 | 0.673072 | 0.989356 | 0.038559 |  | - | - | - | - | - |
| FOSB | 0.056582 | 1.058214 | 1.001429 | 1.118219 | 0.044356 |  | - | - | - | - | - |
| FUT8 | -0.19751 | 0.820775 | 0.708438 | 0.950924 | 0.008537 |  | - | - | - | - | - |
| FZD1 | 0.200509 | 1.222025 | 1.064739 | 1.402544 | 0.00434 |  | - | - | - | - | - |
| GALNT6 | -0.16437 | 0.848429 | 0.749227 | 0.960767 | 0.009575 |  | - | - | - | - | - |
| GAS1 | 0.148486 | 1.160076 | 1.05158 | 1.279766 | 0.003038 |  | 0.187624 | 1.20638 | 1.095226 | 1.328815 | 0.000142 |
| GCH1 | -0.19731 | 0.820934 | 0.710287 | 0.948817 | 0.007557 |  | - | - | - | - | - |
| GFPT2 | 0.117758 | 1.124972 | 1.020289 | 1.240396 | 0.018127 |  | - | - | - | - | - |
| GPS1 | -0.25163 | 0.77753 | 0.611861 | 0.988055 | 0.039565 |  | - | - | - | - | - |
| H6PD | 0.254618 | 1.289969 | 1.095145 | 1.519453 | 0.002304 |  | - | - | - | - | - |
| HDAC4 | 0.229332 | 1.257759 | 1.044831 | 1.51408 | 0.015376 |  | - | - | - | - | - |
| HECA | 0.210619 | 1.234442 | 1.006899 | 1.513406 | 0.042755 |  | - | - | - | - | - |
| HERC1 | 0.3778 | 1.459072 | 1.132401 | 1.87998 | 0.003484 |  | - | - | - | - | - |
| HIBCH | -0.28631 | 0.751027 | 0.597572 | 0.943889 | 0.014083 |  | - | - | - | - | - |
| HIST1H2BK | -0.10882 | 0.89689 | 0.806343 | 0.997604 | 0.045054 |  | - | - | - | - | - |
| HSPG2 | 0.134176 | 1.143594 | 1.018329 | 1.284269 | 0.023402 |  | - | - | - | - | - |
| HTRA1 | 0.125487 | 1.133701 | 1.001104 | 1.283861 | 0.048003 |  | - | - | - | - | - |
| IFI27 | -0.07257 | 0.929998 | 0.872946 | 0.99078 | 0.024657 |  | - | - | - | - | - |
| IFI6 | -0.07872 | 0.924298 | 0.861387 | 0.991804 | 0.028613 |  | - | - | - | - | - |
| IGFBP4 | 0.169435 | 1.184636 | 1.031363 | 1.360686 | 0.016538 |  | - | - | - | - | - |
| IRS1 | 0.156896 | 1.169874 | 1.030251 | 1.32842 | 0.015539 |  | - | - | - | - | - |
| JUNB | 0.167316 | 1.182127 | 1.048729 | 1.332493 | 0.006167 |  | - | - | - | - | - |
| JUP | 0.277852 | 1.320291 | 1.077188 | 1.618259 | 0.007449 |  | - | - | - | - | - |
| KIAA0355 | 0.259123 | 1.295794 | 1.065203 | 1.576302 | 0.009549 |  | - | - | - | - | - |
| KIAA1109 | 0.213729 | 1.238288 | 1.00493 | 1.525834 | 0.044843 |  | - | - | - | - | - |
| KLHDC3 | -0.25377 | 0.775873 | 0.637487 | 0.944301 | 0.01135 |  | - | - | - | - | - |
| LAMP3 | -0.12089 | 0.886128 | 0.816796 | 0.961346 | 0.003634 |  | - | - | - | - | - |
| LTA4H | 0.212025 | 1.236179 | 1.016165 | 1.503828 | 0.033978 |  | - | - | - | - | - |
| LYVE1 | 0.120282 | 1.127815 | 1.03243 | 1.232012 | 0.007634 |  | - | - | - | - | - |
| MBD2 | -0.25797 | 0.772622 | 0.627312 | 0.951591 | 0.015235 |  | - | - | - | - | - |
| MCM3 | -0.24708 | 0.781075 | 0.649924 | 0.938692 | 0.008425 |  | - | - | - | - | - |
| MPHOSPH6 | -0.39597 | 0.673025 | 0.525983 | 0.861173 | 0.001642 |  | - | - | - | - | - |
| MRC1 | 0.101425 | 1.106746 | 1.004803 | 1.219033 | 0.039672 |  | - | - | - | - | - |
| MRPL2 | -0.21991 | 0.80259 | 0.669003 | 0.962852 | 0.017908 |  | - | - | - | - | - |
| NBR1 | 0.248541 | 1.282153 | 1.000201 | 1.643587 | 0.049815 |  | - | - | - | - | - |
| NDNF | 0.091142 | 1.095424 | 1.02296 | 1.173021 | 0.009053 |  | - | - | - | - | - |
| NDUFA5 | -0.24074 | 0.786044 | 0.621184 | 0.994657 | 0.045009 |  | - | - | - | - | - |
| NDUFV2 | -0.24793 | 0.780414 | 0.655407 | 0.929263 | 0.005375 |  | - | - | - | - | - |
| NOTCH2NLA | 0.200096 | 1.221521 | 1.034177 | 1.442802 | 0.018494 |  | - | - | - | - | - |
| NUAK1 | 0.133636 | 1.142977 | 1.022609 | 1.277513 | 0.018586 |  | - | - | - | - | - |
| OARD1 | -0.22777 | 0.796304 | 0.637602 | 0.994507 | 0.044586 |  | - | - | - | - | - |
| OGA | 0.303031 | 1.353957 | 1.055027 | 1.737585 | 0.017274 |  | - | - | - | - | - |
| OGN | 0.090659 | 1.094896 | 1.026506 | 1.167843 | 0.005871 |  | - | - | - | - | - |
| PBK | -0.11814 | 0.888573 | 0.795141 | 0.992983 | 0.037143 |  | - | - | - | - | - |
| PDGFRA | 0.086224 | 1.09005 | 1.001273 | 1.186699 | 0.046667 |  | - | - | - | - | - |
| PDIA4 | -0.24661 | 0.781442 | 0.645973 | 0.945321 | 0.011122 |  | - | - | - | - | - |
| PJA2 | 0.295463 | 1.343749 | 1.036136 | 1.742686 | 0.025907 |  | - | - | - | - | - |
| PPFIA1 | 0.278475 | 1.321114 | 1.025977 | 1.701151 | 0.030868 |  | - | - | - | - | - |
| PPL | 0.194313 | 1.214477 | 1.071676 | 1.376306 | 0.00233 |  | 0.031851 | 1.032364 | 0.900438 | 1.183618 | 0.647968 |
| PSMB9 | -0.09594 | 0.908519 | 0.837013 | 0.986132 | 0.021799 |  | - | - | - | - | - |
| PTGIS | 0.093477 | 1.097986 | 1.016835 | 1.185613 | 0.017027 |  | - | - | - | - | - |
| PTPRU | 0.179995 | 1.197212 | 1.060089 | 1.352072 | 0.00373 |  | - | - | - | - | - |
| RAP1A | -0.33742 | 0.71361 | 0.543615 | 0.936766 | 0.015079 |  | - | - | - | - | - |
| RARRES1 | 0.064643 | 1.066778 | 1.00207 | 1.135665 | 0.042895 |  | - | - | - | - | - |
| RB1 | 0.210246 | 1.233981 | 1.053295 | 1.445663 | 0.009248 |  | 0.192598 | 1.212395 | 1.020836 | 1.4399 | 0.028165 |
| RCBTB2 | 0.166434 | 1.181086 | 1.002668 | 1.391252 | 0.046387 |  | - | - | - | - | - |
| SACS | 0.197778 | 1.218692 | 1.00164 | 1.482778 | 0.048117 |  | - | - | - | - | - |
| SCN3B | -0.08555 | 0.918004 | 0.845179 | 0.997105 | 0.042487 |  | - | - | - | - | - |
| SEC22B | -0.43695 | 0.646006 | 0.475401 | 0.877835 | 0.005226 |  | - | - | - | - | - |
| SEH1L | -0.29726 | 0.742851 | 0.579984 | 0.951454 | 0.01857 |  | - | - | - | - | - |
| SERPINB9 | -0.18307 | 0.832713 | 0.69968 | 0.99104 | 0.039273 |  | - | - | - | - | - |
| SFRP1 | 0.069487 | 1.071959 | 1.009913 | 1.137816 | 0.022359 |  | - | - | - | - | - |
| SHQ1 | -0.32535 | 0.722275 | 0.541143 | 0.964034 | 0.027201 |  | - | - | - | - | - |
| SIRPA | 0.146727 | 1.158038 | 1.0178 | 1.317599 | 0.02589 |  | - | - | - | - | - |
| SLC35A1 | -0.21232 | 0.808707 | 0.673099 | 0.971635 | 0.023376 |  | - | - | - | - | - |
| SPCS2 | -0.22121 | 0.801548 | 0.643267 | 0.998776 | 0.048738 |  | - | - | - | - | - |
| SPG11 | 0.300793 | 1.35093 | 1.052969 | 1.733206 | 0.017984 |  | - | - | - | - | - |
| SPOCK2 | 0.099721 | 1.104863 | 1.026 | 1.189787 | 0.008308 |  | - | - | - | - | - |
| STAB1 | 0.136111 | 1.14581 | 1.023313 | 1.28297 | 0.018302 |  | - | - | - | - | - |
| TBC1D2B | 0.212098 | 1.23627 | 1.031721 | 1.481372 | 0.021541 |  | - | - | - | - | - |
| TBP | 0.264964 | 1.303384 | 1.000757 | 1.697524 | 0.049347 |  | - | - | - | - | - |
| TCEAL4 | -0.24483 | 0.782837 | 0.614563 | 0.997187 | 0.047393 |  | - | - | - | - | - |
| TDP2 | -0.43849 | 0.645012 | 0.494812 | 0.840805 | 0.001187 |  | - | - | - | - | - |
| TGFBR2 | 0.200008 | 1.221413 | 1.023323 | 1.457849 | 0.026738 |  | - | - | - | - | - |
| TGOLN2 | 0.290194 | 1.336687 | 1.030815 | 1.73332 | 0.028605 |  | - | - | - | - | - |
| THY1 | 0.104786 | 1.110473 | 1.008242 | 1.22307 | 0.033459 |  | - | - | - | - | - |
| TMED10 | -0.27187 | 0.761954 | 0.59282 | 0.979342 | 0.033757 |  | - | - | - | - | - |
| TNS3 | 0.138501 | 1.148551 | 1.000473 | 1.318546 | 0.04922 |  | - | - | - | - | - |
| TRA2B | -0.44032 | 0.64383 | 0.443611 | 0.934415 | 0.020509 |  | - | - | - | - | - |
| TRMT1L | -0.26944 | 0.763806 | 0.599859 | 0.972561 | 0.028841 |  | - | - | - | - | - |
| TSPAN13 | -0.15058 | 0.860213 | 0.761765 | 0.971384 | 0.015176 |  | - | - | - | - | - |
| TSPYL1 | 0.230058 | 1.258673 | 1.006619 | 1.57384 | 0.043609 |  | - | - | - | - | - |
| VPS13B | 0.303058 | 1.353993 | 1.096698 | 1.67165 | 0.004827 |  | - | - | - | - | - |
| VSIG4 | 0.119975 | 1.127469 | 1.026527 | 1.238337 | 0.012174 |  | - | - | - | - | - |
| VWA8 | 0.300982 | 1.351185 | 1.094401 | 1.668221 | 0.00513 |  | - | - | - | - | - |
| WTAP | 0.277844 | 1.32028 | 1.028158 | 1.695399 | 0.029435 |  | - | - | - | - | - |
| ZER1 | 0.326582 | 1.386221 | 1.079703 | 1.779757 | 0.010424 |  | - | - | - | - | - |
| ZNF106 | 0.301786 | 1.352271 | 1.077552 | 1.697029 | 0.009198 |  | - | - | - | - | - |
| ZWINT | -0.17106 | 0.84277 | 0.719411 | 0.987283 | 0.034136 |  | - | - | - | - | - |
